# Relationship between in utero sonographic evaluation and subcutaneous plicometry after birth in infants with intrauterine growth restriction: an exploratory study

**DOI:** 10.1186/1824-7288-36-70

**Published:** 2010-10-26

**Authors:** Nadia Liotto, Tatjana Radaelli, Anna Orsi, Emanuela Taricco, Paola Roggero, Maria L Giannì, Dario Consonni, Fabio Mosca, Irene Cetin

**Affiliations:** 1 NICU, Fondazione IRCCS "Ospedale Maggiore Policlinico, Mangiagalli e Regina Elena", University Department of Mother and Infant Sciences, University of Milan, Italy; 2 Institute of Obstetrics and Gynecology "L. Mangiagalli", University of Milan, Fondazione IRCCS Policlinico, Mangiagalli and Regina Elena, Milan, Italy; 3 Center for Fetal Research Giorgio Pardi, University of Milan, Italy; 4 Department of Clinical Sciences, Ospedale Luigi Sacco, University of Milan, Milan, Italy; 5 Unit of Epidemiology, Department of Preventive Medicine, Fondazione IRCCS Ospedale Maggiore Policlinico, Mangiagalli e Regina Elena, Milan, Italy

## Abstract

**Background:**

Intrauterine growth restriction (IUGR) is associated with several medical complications before and after delivery. The aim of this study was to evaluate the concordance between the fetal ultrasonographic measurement of subcutaneous tissue thicknesses and the skinfold thicknesses assessment in intrauterine growth restricted newborns.

**Methods:**

We designed an exploratory study. Fetal ultrasonographic measurement of subcutaneous tissue thicknesses, according to Bernstein's and Galan's method, and neonatal skinfold thicknesses were evaluated in 13 intrauterine growth restricted newborns within 4 hours before delivery and on the first day of life, respectively. Concordance between fetal and neonatal measurements was assessed using the Lin's correlation coefficient and the Bland-Altman method.

**Results:**

The data obtained by the measurements of neonatal skinfold thicknesses was significantly correlated with the prenatal measurements (Lin's coefficients, arm: 0.60; subscapular: 0.72; abdomen: 0.51). Bland-Altman analysis showed moderate agreement between the fetal ultrasonographic measurement of subcutaneous tissue thicknesses and the neonatal skinfold thicknesses assessment.

**Conclusions:**

The present study provides preliminary evidence that fetal sonographic measurements may represent additional indices of intrauterine growth restriction.

## Introduction

Intrauterine growth restriction (IUGR) is associated with several prenatal and postnatal complications and it increases the risks of cardiovascular and metabolic diseases in adulthood [1-3]. Early diagnosis of IUGR is therefore advisable.

Serial prenatal ultrasound measurements have been proposed to monitor the occurrence of IUGR. Indeed, estimated fetal weight is commonly used as an index of fetal growth, although this method presents some limitations [4]. Measurements of the abdominal circumference at the level of the fetal liver is currently considered as an indicator of intra-uterine fetal growth in the second half of pregnancy [5]. The rationale for this measurement is that it corresponds most closely with the size of the fetal liver as well as to fetal fat deposition. Subcutaneous thicknesses have been proposed as measurements of fat in different areas of the fetus in addition to the routine ultrasound-derived biometric parameters in different intrauterine growth conditions [6-9].

We designed an exploratory study to evaluate the concordance between the fetal ultrasonographic measurement of subcutaneous tissue thicknesses and the skinfold thicknesses assessment in intrauterine growth restricted newborns.

## Methods

### Subjects

Thirteen caucasian pregnant women were enrolled for the study among the pregnant patients followed from January to December 2007 at the Author's Institution. Inclusion criteria were presence of IUGR and singleton pregnancy. Exclusion criteria were: gestational diabetes, smoking, alcohol abuse, drug addiction, abnormal fetal karyotype, fetal malformations and infections. Gestational age was calculated from the last menstrual period and confirmed by routine ultrasonography at 11-13 weeks of gestation [10].

IUGR was defined by the occurrence of a fetal abdominal circumference below the 10th centile of reference values for fetuses of similar ages and a decrease of more than 40 percentiles from the age specific size curve [11].The study was approved by the local Institutional Review Board. Informed consent was obtained from all pregnant women prior to the beginning of the study.

#### In utero ultrasound evaluation

**Figure 1:**
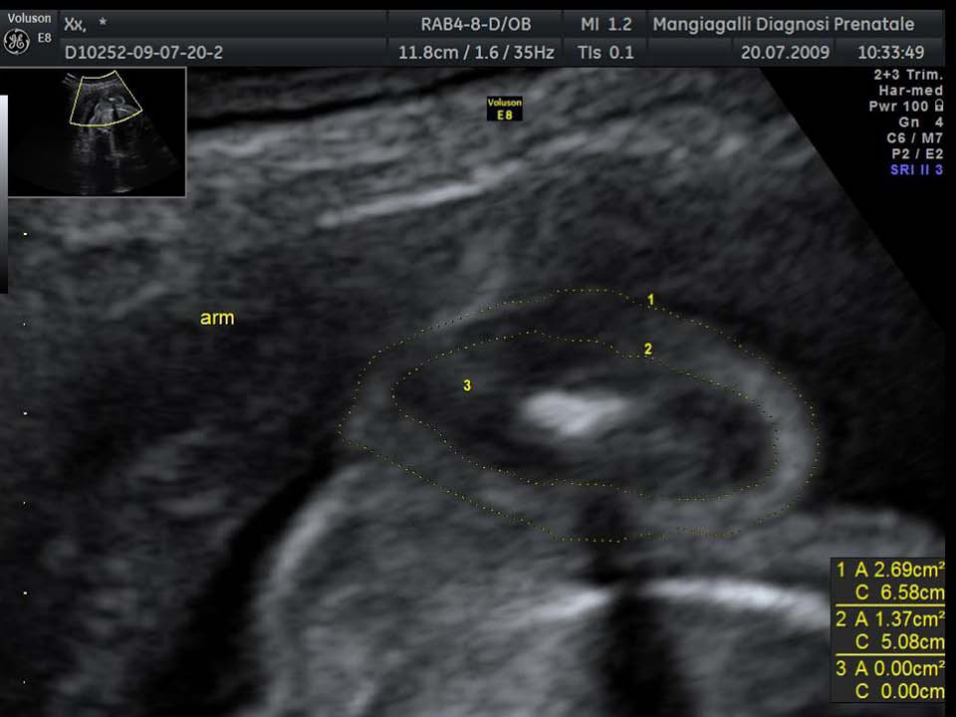
**Upper arm fat (grey arrow) and lean (white arrow) areas**. Fat area was measured by taking the total cross-sectional limb area and subtracting the central lean area consisting of muscle and bone.

A fetal ultrasound evaluation was performed within 4 hours before the delivery. A 5 MHz convex probe was used for all measurements (Voluson 730 Expert-GE Medical Systems). During the examination, routine ultrasonographic biometric parameters including head circumference, bi-parietal diameter, abdominal circumference and femur length were obtained together with a more complex evaluation of fetal fat mass. Ultrasound measurements of fat were obtained on cross-sectional images of the proximal arm and the abdomen as previously described by Bernstein and colleagues [12] and more recently by Galan and colleagues [8]. A longitudinal view of the long bone was obtained and used to identify the midpoint of the arm. The transducer was then rotated 90° to obtain the cross-sectional view of the mid-limb. Fat body mass area of the mid upper arm was measured by taking the total cross-sectional limb area and subtracting the central lean area consisting of muscle and bone on axial ultrasound images (Figure 1).

**Figure 2:**
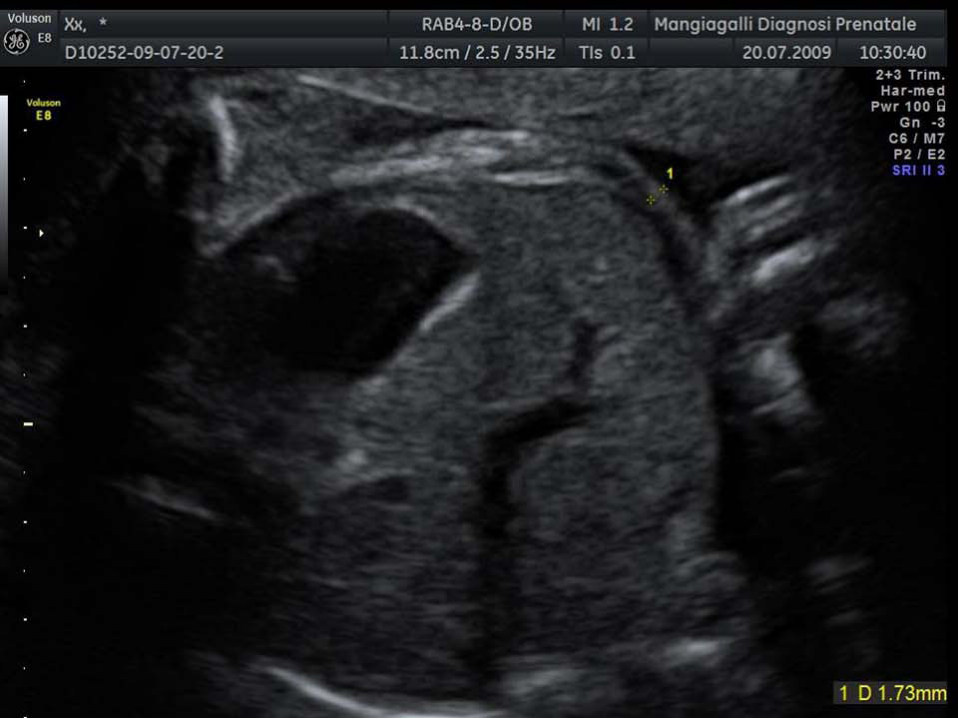
**Abdominal subcutaneous tissue thickness**. The fat thickness was determined by measuring the thickness of the anterior abdominal subcutaneous tissue on the same axial image on which the abdominal circumference was obtained and positioning the caliper on the proximal midaxillary line: arrow indicates site where measurement was taken.

The fat mass of the abdomen was determined by measuring the thickness of the anterior abdominal subcutaneous tissue on the same axial image on which the abdominal circumference was obtained[13] and positioning the caliper on the proximal midaxillary line (Figure 2).

**Figure 3:**
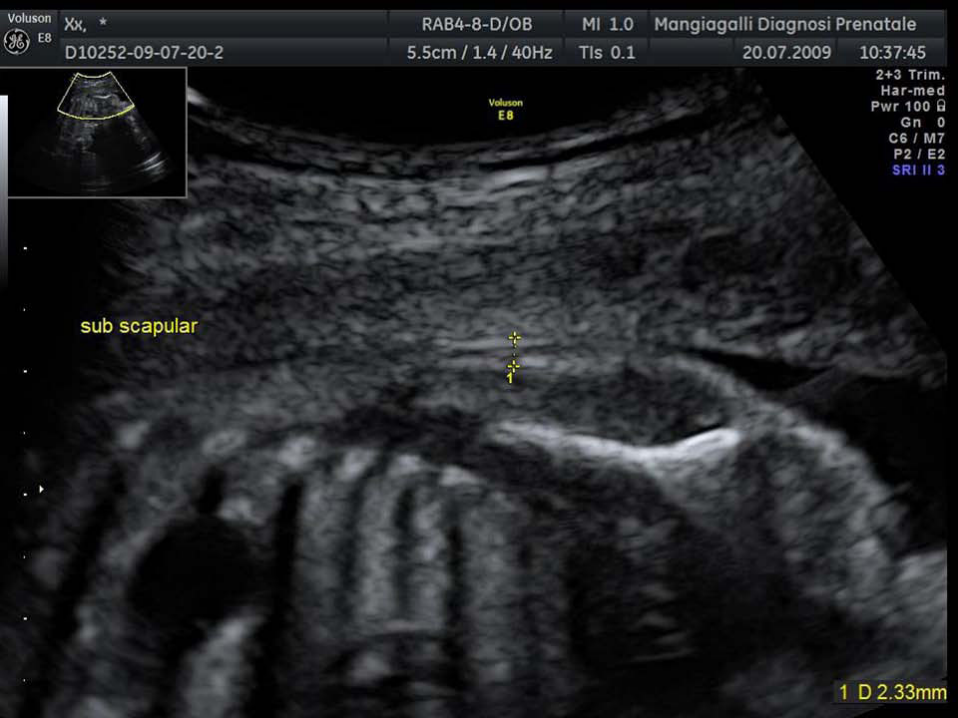
**Subscapular fat thickness**. The fat thickness was evaluated longitudinally on the fetal trunk, visualizing the entire scapula, between the skin surface and the subcutaneous tissue at the interface with the super-and infra-spinous muscles: arrow indicates site where measurement was taken.

Subscapular fat thickness was evaluated longitudinally on the fetal trunk, visualizing the entire scapula, positioning the caliper between the skin surface and the subcutaneous tissue at the interface with the super- and infra-spinous muscles (Figure 3) [7].

Two measurements were made for each of these parameters and the mean value was used in the analysis. All the measurements were performed by the same trained operator. The intraobserver coefficient of variation for the abdominal proximal arm and subscapular subcutaneous thickness was 2.6%, 2%, 2.1%, respectively (unpublished observations).

Mothers' age, pre-pregnancy weight, height and BMI (kg/m2) and weight increase during pregnancy were collected as maternal variables. The placental weight was also recorded.

#### Neonatal anthropometric measurements

Anthropometric measurements (weight, length and head circumference) and subcutaneous skinfold thicknesses were assessed in all infants on the first day of life by the same trained operator. The investigator who performed the postnatal measurements was blinded to the antenatal results.

Body weight, length and head circumference were measured according to standard procedures [14]. Babies weight was measured on an electronic scale accurate to ±5 g (Seca scale, Intermed s.r.l. San Giuliano Milanese, Milano, Italy) and body length was measured to the nearest millimeter on a Harpenden neonatometer (Holtain Ltd, UK). Head circumference was measured to the nearest millimeter with non-stretch measuring tape.

Left skinfold thicknesses were measured using a commercial caliper (Harpenden Skinfold Caliper, Baty International, West Sussex, UK) at the following sites: biceps, triceps, subscapular and suprailiac. Skinfold thicknesses were assessed three times and the mean of three readings was taken.

The skinfolds were measured by elevating a fold of skin and subcutaneous tissue between the operator's thumb and index, from the underlying muscle tissue [15,16]. The intra-observer repeatability was 0,20 mm. Triceps skinfolds were measured at the level of the mid-arm circumferences between the acromion and the olcranon processes. Biceps was measured at the same level but on the anterior arm's surface. Immediately below the lower angle of the scapula, subscapular skinfold was measured. Suprailiac skinfold was measured immediately above the iliac crest, 1 cm towards the medial line. In order to account for the fluctuation in total body water that occurs during the early days of life, dynamic skinfold thickness was obtained by reading each skinfold thickeness after a 60 second pressure [17]. All measurements were to the nearest mm.

### Statistical analysis

Descriptive data are expressed as mean (SD) or number of observations (percentage).

Concordance between fetal and neonatal measurements were evaluated using Lin's correlation coefficient [18,19] and the Bland-Altman method [20,21]. Data obtained from the ultrasonographic measurement of the upper arm was compared with the value obtained by the sum of biceps and triceps skinfold thicknesses [22]. Data obtained from the ultrasonographic measurement of the abdomen was compared with suprailiac skinfold thickness, and data obtained from the ultrasonographic measurement of the subscapular region was compared with subscapular skinfold thickness.

Statistical significance was set at 0.05 level. Statistical analyses were performed using Stata, Version 11 [23].

## Results

Ultrasonographic subcutaneous tissue thicknesses, postnatal anthropometric parameters and skinfold thicknesses were assessed in all the mother-infant pairs enrolled in the study.

The maternal and neonatal characteristics of each mother-newborn pair studied are presented in Table 1 and Table 2. Mean maternal age (y) was 32.9 (4.5). The mean pre-pregnancy weight (Kg) and BMI (kg/m2) were 60.5 (10) and 22.7 (3.8), respectively. The mean weight increase during pregnancy was 10 (3) kg.

**Table 1:** Maternal characteristics

n°	Age (y)	Weight (kg)	Height (m)	BMI **(Weight/h**^**2**^**)**	Weight Increase (kg)	Clinical history
1	39	58	1.73	19.4	6	
2	40	50	1.58	20.0	11	
3	35	57	1.71	19.5	14	*Preeclampsia*
4	17	60	1.65	22.0	-5	
5	37	60	1.65	22.0	10	
6	33	77	1.55	32.1	1	
7	27	65	1.68	23.0	8	
8	37	55	1.62	20.1	20	
9	34	72	1.70	24.9	5	
10	36	52	1.67	18.7	12	*Anorexia since 19 to 27 y*
11	40	53	1.65	19.5	9	*Uterine polyposis*
12	33	58	1.63	21.8	7	
13	33	59	1.65	21.7	9	

**Table 2:** Neonatal characteristics

n°	Gender	Gestational age at delivery (wks)	IUGR diagnosis (wks)	Birth weight (g)	Neonatal Length (cm)	Head Circumference (cm)	Apgar score (1'-5')	Placental weight (g)
1	F	35	28	1460	43.0	31.5	9/10	280
2	M	36	35	1890	44.3	31.1	9/9	320
3	F	38	37	2620	44.0	31.5	9/10	280
4	M	36	34	1650	44.8	33.0	8/9	240
5	F	38	38	1950	40.0	30.0	9/10	275
6	M	31	28	1050	40.5	29.3	9/10	265
7	F	34	21	1540	41.1	30.5	8/9	220
8	F	32	28	1160	40.5	31.0	9/10	280
9	M	35	34	1500	43.0	32.5	8/9	195
10	F	32	27	1180	44.0	32.0	8/9	180
11	M	34	32	1590	37.0	32.0	7/9	230
12	F	33	33	1450	37.0	31.0	7/9	229
13	M	33	31	1240	43	32.5	8/9	260

The occurrence of IUGR was diagnosed at an average gestational age of 31.5 (5.12) weeks. The umbilical artery Doppler was abnormal in six women. Maternal anorexia, uterine polyposis and preeclampsia were recorded in three cases. The remaining pregnancies with IUGR were apparently not complicated by other maternal, placental or fetal pathologies. The mean placental weight was 250 (39) g.

All infants were delivered by caesarean section, performed in the interest of the fetus.

The mean gestational age at delivery was 35 (2.4) weeks. The mean neonatal birth weight, length and head circumference were 1560 (417) g, 42.9 (1.7) cm and 31.2 (1.1) cm, respectively. None of the infants died during hospital stay. Respiratory support was needed in four infants.

A significant concordance correlation coefficient (0.60, p = 0.006) was found between the value obtained by the sum of biceps and triceps skinfold thicknesses and the ultrasonographic measurement of the fat area of the upper arm. Neonatal subscapular skinfold thickness significantly correlated (0.72; p = 0.002) with ultrasonographic measurement of fat in the subscapular region. Neonatal suprailiac skinfold thickness showed a significant correlation (0.51; p = 0.03) with ultrasound measurement of fat at the level of the abdomen.

**Figure 4:**
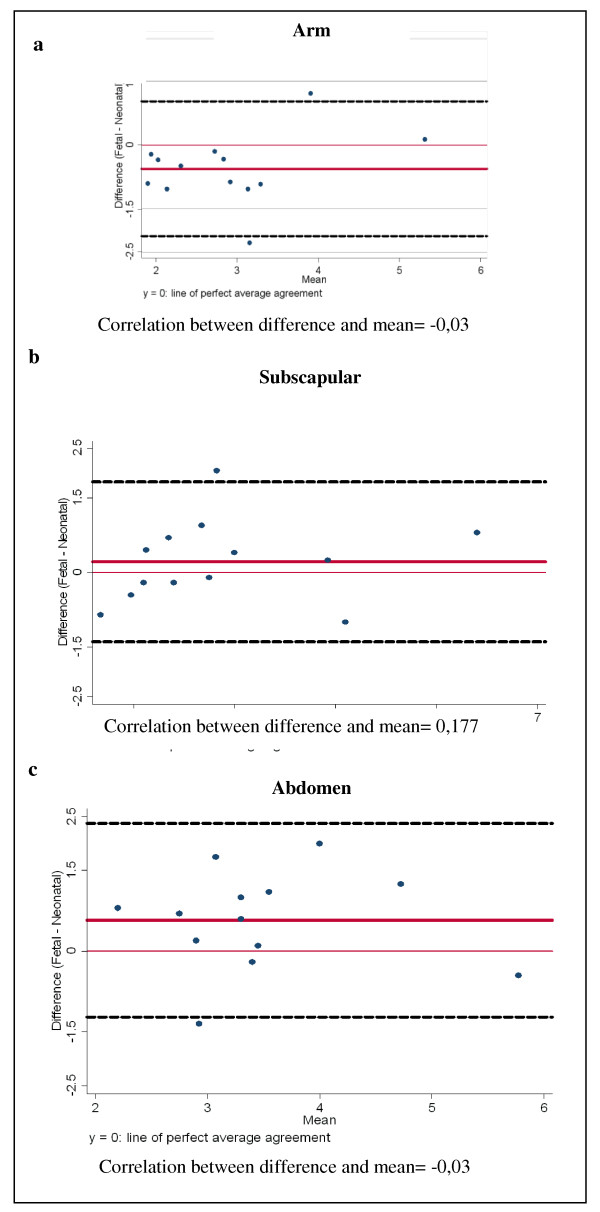
**Concordance between fetal ultrasonographic measurement of subcutaneous tissue thicknesses and neonatal skinfold thicknesses assessment according to Bland Altman method (--- 95% limits of agreement)**. Figure a: Ultrasonographic measurement of the fat area of the upper arm (cm2) and the sum of biceps and triceps skinfold thickness (cm). Figure b: Ultrasonographic measurement of fat in the subscapular region (cm) and neonatal subscapular skinfold thickness (cm). Figure c: Ultrasound measurement of abdominal fat (cm) and neonatal suprailiac skinfold thickness (cm).

Results of Bland Altman analysis are shown in figure 4. The ultrasonographic measurement of the fat area of the upper arm underestimated [average difference = -0.6 cm2 (SD = 0.8; 95% Limits of Agreement -2.1; 1.0)] the sum of biceps and triceps skinfold thicknesses in newborns. Fetal subscapular skinfold thickness underestimated neonatal subscapular fat of 0.2 cm (SD = 0.8; 95% Limits of Agreement -1.4; 1.8). Fetal abdominal thickness underestimated neonatal suprailiac fat of 0.5 cm (SD = 0.9; 95% Limits of Agreement -1.2; 2.3).

## Discussion

The present study demonstrates a moderate concordance between fetal and neonatal measurements in IUGR.

Several authors have proposed different methods to evaluate intrauterine growth restriction. In particular, estimated fetal weight is commonly used as an index of fetal growth and is generally calculated through a combination of parameters that include, amongst others, the abdominal circumference [4]. Errors in estimated fetal weight could be as high as 25% [5] and result from technical measurement errors, as well as the assumptions that fetal density is constant throughout gestation and independent of the fetal nutritional and endocrine processes that can alter the normal ratios of muscle and fat [8,12].

The correlation between birth weight and the occurrence of growth restriction has been investigated by means of serial ultrasonographic evaluations. Anusha and coworkers [24] showed that birth weight is not a good indicator of fetal growth restriction and suggested that fat mass could be a better indicator. Indeed, fat mass constitutes 12-14% of birth weight and has been shown to account for 46% of the variation in neonatal weight [4]. Consequently, ultrasound-generated estimates of fetal fat mass may be useful in the evaluation of fetal growth abnormalities.

Lee and al. [25] proposed the use of tridimensional ultrasonographic methods to evaluate fetal growth and the amount of soft tissue during the third trimester in relation to birth weight and the neonatal body composition performed by means of a pediatric air displacement plethysmography system. They described that percentage of neonatal body fat is correlated to thigh volume, improving the correlation obtained by abdominal circumference or estimated fetal weight alone. Our data are in agreement with the data of Lee and suggest the usefulness of a simple assessment tool that does not require the availability of a 3D instrument.

Growth restriction plays a key role in the later development of diseases, such as type II diabetes, cardiovascular diseases, hypertension, obesity and neural-developmental deficits, not only in the immediate postnatal period but also later in life [1,26,27].

Consequently, early diagnosis of IUGR is advisable in order to initiate appropriate nutritional strategies and prevent the development of later complications Furthermore early diagnosis is extremely useful in the detection of the optimal timing for delivery. Indeed, changing from an unfavorable intrauterine environment and implementing nutritional procedures allow IUGR infants to decrease their risk of developing the metabolic syndrome [28].

Although the present study is an exploratory study investigating the concordance between the measurements performed by means of sonographic evaluations and neonatal plicometry, it provides preliminary evidence that sonographic methods for the measurement of fetal fat may represent additional indices of intrauterine growth restriction. As the clinical implication of these results could positively affect the management of these infants, further larger studies are desirable.

## Competing interests

The authors declare that they have no conflict of interest in connection with this paper.

## Authors' contributions

All the authors participated in the concept, design and revision of the manuscript and approved the final version.
